# Efficacy and safety of immune checkpoint inhibitors in Proficient Mismatch Repair (pMMR)/ Non-Microsatellite Instability-High (non-MSI-H) metastatic colorectal cancer: a study based on 39 cohorts incorporating 1723 patients

**DOI:** 10.1186/s12865-023-00564-1

**Published:** 2023-09-01

**Authors:** Qing Wu, Ziming Wang, Yang Luo, Xianhe Xie

**Affiliations:** 1 Department of Oncology, Molecular Oncology Research Institute, The First Affiliated Hospital of Fujian Medical University, Fuzhou, 350005 China; 2 Department of Oncology, National Regional Medical Center, Binhai Campus of The First Affiliated Hospital, Fujian Medical University, Fuzhou, 350212 China; 3 Fujian Key Laboratory of Precision Medicine for Cancer, The First Affiliated Hospital of Fujian Medical University, Fuzhou, 350005 China

**Keywords:** Immune checkpoint inhibitors, pMMR, Non-MSI-H, Colorectal cancer, Efficacy, Safety

## Abstract

**Purpose:**

This study was designed to investigate the efficacy and safety of immune checkpoint inhibitors (ICIs)-based therapy in proficient mismatch repair (pMMR)/non-microsatellite instability-high (non-MSI-H) metastatic colorectal cancer (mCRC).

**Methods:**

Electronic databases were screened to identify relevant trials. The primary endpoints were pooled objective response rate (ORR) and disease control rate (DCR). Stratified analysis was accomplished on ICIs-based regimens, treatment lines and RAS status.

**Results:**

Totally, 1723 mCRC patients from 39 cohorts were included. The pooled ORR, DCR, 12-month overall survival (OS) rate and 6-month progression-free survival (PFS) rate of ICIs-based therapy in pMMR/non-MSI-H mCRC were 8.5% (95% CI: 4.4%-13.5%), 48.2% (95% CI: 37.8%-58.6%), 52.3% (95% CI: 46.4%-58.1%) and 32.8% (95% CI: 23.5%-42.7%) respectively. As a whole, no significantly differences were shown between ICIs-based and non-ICIs-based therapy for pMMR/non-MSI-H mCRC in terms of both PFS (HR = 1.0, 95% CI: 0.9–1.1, *P* = 0.91) and OS (HR = 1.0, 95% CI: 0.9–1.2, *P* = 0.51). It was worth noting that the addition of ICIs to anti-vascular endothelial growth factor (VEGF) agent plus chemotherapy displayed excellent efficacy in pMMR/non-MSI-H mCRC (ORR = 42.4%, 95% CI: 10.0%-78.6%; DCR = 92.0%, 95% CI: 68.3%-100.0%; 12-month OS rate = 71.4%, 95% CI: 50.0%-89.1%; 6-month PFS rate = 55.2%, 95% CI: 24.8%-83.8%; and PFS (compared with non-ICIs-based therapy): HR = 0.9, 95% CI: 0.8–1.0, *P* = 0.02), especially served as first-line therapy (ORR = 74.2%, 95% CI: 61.4%-85.4%; DCR = 98.7%, 95% CI: 92.0%-100.0%); and without additional treatment related adverse events (TRAEs) were observed.

**Conclusions:**

ICIs-based combination therapy, especially the addition of ICIs to first-line anti-VEGF agent plus chemotherapy, is promising in pMMR/non-MSI-H mCRC with good efficacy and controllable toxicity.

**Supplementary Information:**

The online version contains supplementary material available at 10.1186/s12865-023-00564-1.

## Introduction

Metastatic colorectal cancer (mCRC) is one of the major causes of cancer-related morbidity and mortality all over the world [1]. Despite remarkable improvements have been made in clinical outcomes with the optimization of chemotherapy and targeted therapy, the results continue to fall far short of durable curative treatment of mCRC patients. Consequently, it is crucial to seek a novel approach against mCRC. During the last decade, immune checkpoint inhibitors (ICIs) have made tremendous breakthroughs in the clinical treatment of several hematological and solid tumors, including Hodgkin lymphoma, malignant melanoma, non-small cell lung cancer (NSCLC), triple negative breast cancer (TNBC), advanced hepatocellular carcinoma (HCC) and microsatellite instability-high (MSI-H) mCRC [2–7]. However, ICIs remain largely ineffective in the majority of mCRC patients, characterized by proficient mismatch repair (pMMR)/non-MSI-H.

It has been recorded that a lack of efficacy of the anti-PD-1 and a modest clinical benefit of the anti-PD-L1 plus the anti-CTLA-4, reserved only to patients with a tumor mutational burden (TMB) more than 28 mut/Mb on circulating tumor DNA [8, 9]. Based on these considerations, accumulating focus has been recently placed on developing effective combination regimens in which ICIs have been incorporated with chemotherapy, radiotherapy and biologic agents with the purpose of reshaping the microenvironment of pMMR/non-MSI-H tumors towards an immune-inflamed/hot phenotype, that may lead to ICIs sensitivity. However, much of these approaches have been largely disappointing [10–12]. Notably enough, two phase II studies named AtezoTRIBE and MAYA, assessing combinations of ICIs with chemotherapy, have rekindled hope for the use of ICIs in pMMR/non-MSI-H mCRC [13, 14].

To overcome the limitations of individual studies and assess the overall benefit, therefore, we conducted a comprehensive survey based on a large sample size (39 cohorts incorporating 1723 individuals), diverse dimensions (including pooled rate, odd ratio (OR), and hazard ratio (HR)), multiple stratifications (based on ICIs-based regimens, treatment lines and RAS status), and various evaluation indicators (incorporating objective response rate (ORR), disease control rate (DCR), progression-free survival (PFS) and overall survival (OS)) to evaluate the efficacy and safety of ICIs-based therapy in pMMR/non-MSI-H mCRC.

## Materials and methods

### Data sources and literature searches

Articles were screened through a systematic electronic literature retrieval for abstracts of relevant studies in the published literature. PubMed, Cochrane Library, and EMBASE were searched and the data were updated as of August 15th, 2022. The basic search terms were used as follows: “immunotherapy”, “immune checkpoint inhibitor”, “Pembrolizumab”, “Atezolizumab”, “Nivolumab”, “PD-1”, “Keytruda”, “Tecentriq”, “Bavencio”, “Imfinzi”, “PD-L1”, “CTLA-4”, “Ipilimumab”, “programmed cell death 1”, “programmed cell death-ligand 1”, “cytotoxic T lymphocyte-associated protein 4”, “ICI”, “Sintilimab”, “Camrelizumab”, “Tislelizumab”, “Durvalumab”, “Avelumab”, “colon cancer”, “colorectal cancer”, “rectal cancer”, “microsatellite instability-low”, “MSI-L”, “MS-S”, “MSS”, “microsatellite stable”, and “pMMR”. Full-text papers were scrutinized if abstracts did not provide substantial information. Moreover, the references of relevant articles were reviewed for additional studies. Data retrieval was accomplished in English.

### Selection of studies

Initially, two investigators performed a screening of titles and abstracts respectively, then examined the full-text of articles to acquire eligible studies. For the duplicate studies based on the same study patients, only the latest or most comprehensive data were recruited.

### Inclusion criteria

(1) Prospective or retrospective studies to evaluate the efficacy and safety of ICIs in pMMR/non-MSI-H mCRC; (2) patients pathologically confirmed as CRC; (3) the data (involving any of the following outcomes: ORR, DCR, PFS, OS, 6-month PFS rate and 12-month OS rate) to evaluate the efficacy of ICIs in pMMR/non-MSI-H mCRC could be obtained or calculated from the original literature.

### Data extraction

Data extraction was implemented conforming to the PRISMA guidance (Table S1). All eligible studies involved information as follows: the first author’s name, publication year, number of pMMR/non-MSI-H mCRC patients, ICIs agent, and endpoints.

### Quality assessment

The quality of included studies was assessed independently by two reviewers using the Newcastle–Ottawa Scale (NOS) for case–control and cohort studies. It encompassed three dimensions of selection, comparability, and exposure, with a full score of 9 points.

### Statistical methods

The primary endpoints were ORR and DCR measured by pooled rates with corresponding 95% CIs for pMMR/non-MSI-H mCRC. The secondary endpoints were pooled PFS, OS, 6-month PFS rate and 12-month OS rate. Subgroup analysis was accomplished based on various ICIs-based regimens, treatment lines and RAS status. The summary measures of prognostic parameters and adverse events (AEs) were pooled rate, ORs and HRs with 95% CIs. Funnel plots and Egger’s test were performed to evaluate publication bias. Statistical analysis was performed with R 4.0 statistical software. Survival data were obtained based on the Kaplan–Meier curves. Heterogeneity was assessed by I-square tests and Chi-square. If *P* < 0.1 or *I*^*2*^ > 50%, remarkable heterogeneity existed. A random effect model was adopted to calculate the pooled data when heterogeneity existed, or else, a fixed effect model was employed.

## Results

### Selection of study

**Fig. 1:**
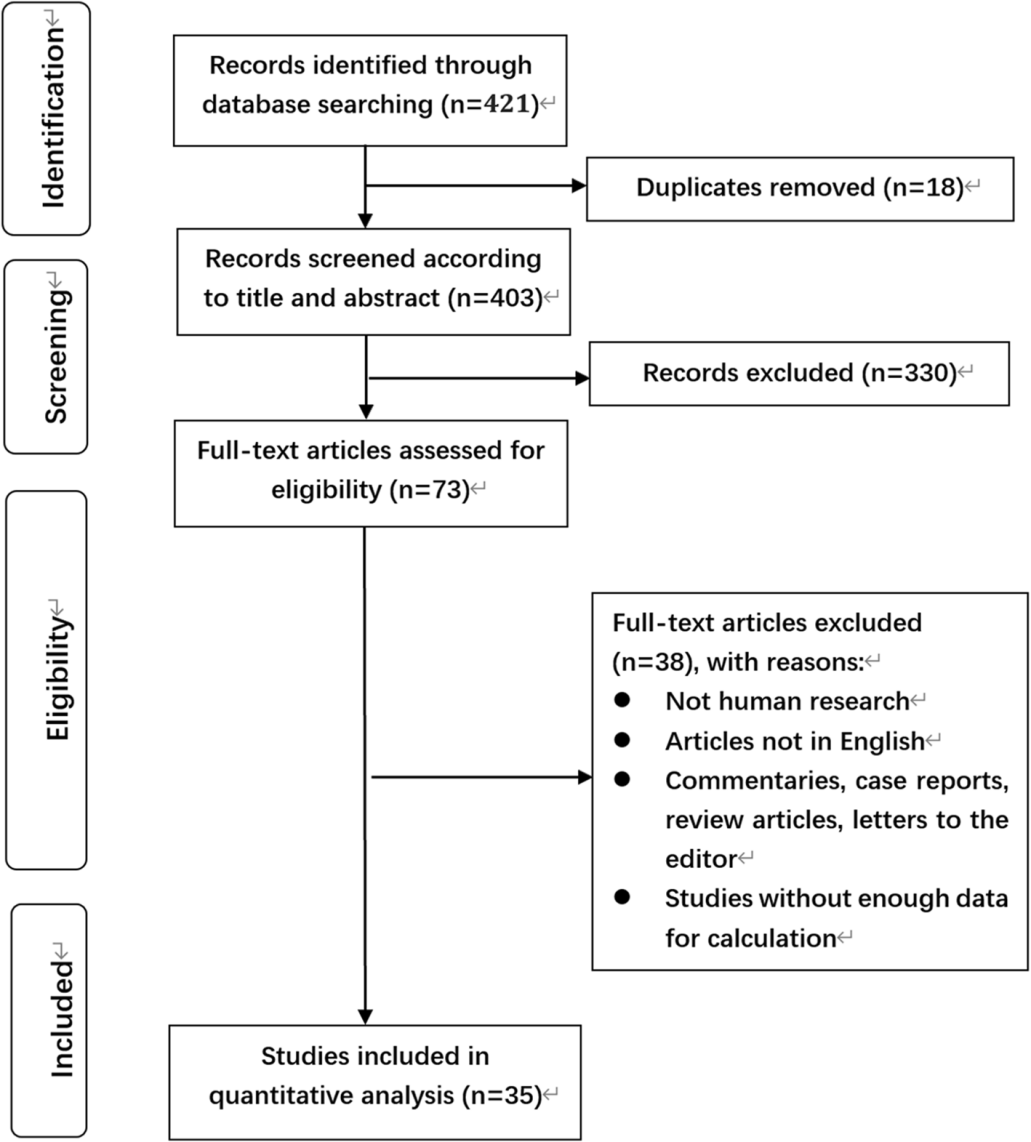
Flowchart on selection including trials in the meta-analysis

Initially, 421 relevant articles were scrutinized intensively. Of them, 18 were filtered for duplication, and 330 were excluded for digression after screening the titles and abstracts. Then the full text of 73 articles was thoroughly reviewed, and 38 were filtered for: they were not human research, and not in English, commentaries, case reports, review articles, or letters to the editor, and without enough data for calculation. Finally, a total of 35 articles (including 39 cohorts) incorporating 1723 patients were recruited in this study (Table S2). The elaborate procedure was displayed in Fig. 1.

### Study traits

Totally, 1723 individuals from the 35 articles (39 cohorts) published until August 15th, 2022 were recruited. The sample size ranged from 6 to 250. Of these studies, 4 were randomized controlled trials (RCTs), and 9 retrospective studies. Meanwhile, all of these studies involved ICIs: ICIs monotherapy, ICIs plus targeted therapy, ICIs plus chemotherapy or radiotherapy, ICIs plus both targeted therapy and chemotherapy/radiotherapy. Pooled rate and 95% CIs were used to report the ORR, DCR, 6-month PFS rate, 12-month OS rate, and AEs of ICIs in pMMR/non-MSI-H mCRC; HRs with corresponding 95% CIs were utilized to assess the PFS and OS of ICIs for pMMR/non-MSI-H mCRC. The principal traits were presented in Table 1.

**Table 1:** The principal characteristics of eligible articles

**First author**	**Year**	**Clinicaltrials.gov**	**Study phase**	**MSI/MMR status**	**No. patients treated with ICIs**	**Treatment line**	**ICIs agent**	**Dose**	**Combination drug**
Gou M [15]	2022	NA	Retrospective, SA	non-MSI-H/pMMR	45	≥ 3	anti-PD-1	Pembro, Sin, Camre: 200 mg; Nivo: 3 mg/kg, q3w	Fruquintinib
Antoniotti C [13]	2022	NCT03721653	RCT, phase II	pMMR	132	1	Atezo	840 mg, q2w	FOLFOXIRI + Bevacizumab
Xu YJ [16]	2022	NA	Retrospective, SA	MSS/pMMR	30	≥ 3	anti-PD-1	Tori: 240 mg q3w, Camre: 200 mg q2 or 3w, Nivo: 240 mg q2w, Pembro, Sin, tislelizumabor: 200 mg q3w	Rego
Morano F [14]	2022	NCT03832621	MC, SA, phase II	MSS	33	≥ 2	IPI and Nivo	IPI: 1 mg/kg q8w, Nivo: 480 mg q4w	Temozolomide
Mettu NB [17]	2022	NCT02873195	RCT, phase II	MSS/pMMR	69	≥ 2	Atezo	840 mg, q2w	Capecitabine + Bevacizumab
Rahma OE [11]	2022	NCT02298959	MC, phase IB	MSS	6	≥ 2	Pembro	2 mg/kg, q2w	Ziv-aflibercept
Kim RD [18]	2022	NCT03712943	OL, SA, phase I/Ib	pMMR	51	≥ 3	Nivo	240 mg	Rego
Redman JM [19]	2022	NCT03050814	RCT, phase II	MSS	16	1	Ave	10 mg/kg q2w	AdCEA Vaccine + mFOLFOX6 + Bevacizumab
Fukuoka S [20]	2020	NCT03406871	OL, phase Ib	MSS/pMMR	25	≥ 3	Nivo	3 mg/kg, q2w	Rego
Eng C [10]	2019	NCT02788279	RCT, MC, OL, phase III	MSS/MSI-L	170	NA	Atezo	840 mg, q2w	Cobimetinib
					83	NA	Atezo	1200 mg, q3w	None
Kawazoe A [21]	2020	NCT02851004	MC, phase I/II	MSS	40	NA	Pembro	200 mg, q3w	Napabucasin
Ren C [22]	2020	NCT03912857	Prospective, SA, OL, phase II	MSS	10	NA	Camre	200 mg, q2w	Apatinib
Kawazoe A [23]	2021	UMIN000032801	OL, phase Ib	MSS	25	≥ 2	Nivo	3 mg/kg, q2w	TAS-116 (Pimitespib)
Parikh AR [12]	2021	NCT03104439	SA, non-randomized, phase II	MSS	27	≥ 2	IPI and Nivo	Nivo: 240 mg and IPI: 1 mg/kg	Radiation
					13	≥ 2			None
Wang C [24]	2020	NA	Retrospective	MSS	18	≥ 3	anti-PD-1	Pembro: 200 mg q3w, Nivo: 240 mg q2w	Rego
Cousin S [25]	2021	NCT03475953	SA, OL, Phase II	MSS	47	≥ 2	Ave	10 mg/kg q2w	Rego
Wang C [26]	2020	NCT03005002	SA, Phase II	MSS	9	≥ 3	Treme + Durva	Treme: 75 mg q4w + Durva: 1500 mg q4w	Yttrium-90 Liver Radioembolization
Li J [27]	2020	NA	Retrospective	MSS/pMMR	23	≥ 3	anti-PD-1	Nivo, Pembro, Camre, Sin, Tori	Rego
Hellmann MD [28]	2019	NCT01988896	MC, OL, phase I/Ib	MSS/MSI-L	62	≥ 1	Atezo	800 mg, q2w	Cobimetinib
Kim DW [29]	2021	NCT03332498	Phase II	pMMR	31	≥ 2	Pembro	200 mg q3w	Ibrutinib
Patel MR [30]	2021	NCT02860546	SA, phase II	MSS	18	≥ 3	Nivo	3 mg/kg, q2w	Trifluridine/tipiracil
Bordonaro R [31]	2021	NCT02848443	OL, MC, phase I	MSS	17	≥ 2	Nivo	3 mg/kg, q2w	Trifluridine/tipiracil (FTD/TPI) + oxaliplatin
Zhou H [32]	2021	NA	Retrospective	MSS/pMMR	21	1	Camre	200 mg q3w	XELOX + Bevacizumab or Rego
Yu W [33]	2021	NA	Retrospective	MSS	33	≥ 3	Tori	240 mg q3w	Rego
Sun L [34]	2021	NA	Retrospective	MSS	23	≥ 4	anti-PD-1	Tori: 240 mg q3w, Nivo: 200 mg q2w, Sin or Camre: 200 mg q3w	Rego
					28	≥ 4			Fruquintinib
Jiang FE [35]	2021	NA	Retrospective	MSS/pMMR	16	≥ 3	Camre	200 mg q3w	Rego or Fruquintinib
O'Neil BH [36]	2017	NCT02054806	MC, phase Ib	MSS	19	≥ 1	Pembro	10 mg/kg q2w	None
Yarchoan M [37]	2020	NCT02981524	SA, phase II	pMMR	17	≥ 3	Pembro	NA	GVAX colon vaccine
Taylor K [38]	2020	NCT02811497	MC, OL, phase II	MSS	15	≥ 4	Durva	1500 mg q3w	CC-486
Martinelli E [39]	2021	NCT04561336	SA, phase II	MSS	71	≥ 3	Ave	10 mg/kg q2w	Cetuximab
Wang C [40]	2021	NA	Retrospective	MSS	95	≥ 3	anti-PD-1/PD-L1	NA	None
Lee JJ [41]	2017	NCT02260440	SA, phase II	MSS	30	≥ 3	Pembro	200 mg q3w	Aza
Fang X [42]	2022	NCT05171660	OL, SA, phase II	MSS	25	1	Sin	200 mg q3w	CapeOx and Bevacizumab
Bocobo AG [43]	2021	NCT03396926	OL, SA, phase II	MSS	29	≥ 2	Pembro	200 mg q3w	Capecitabine and Bevacizumab
Huyghe N [44]	2022	NCT03608046	Phase I	MSS	10	≥ 3	Ave	10 mg/kg q2w	Cetuximab and Irinotecan
					13	≥ 3			
**First author**	**Male**	**median age (range)**	**Median follow-up time (95%CI)**	**No. of control**		**Control**	**Endpoints**	**median PFS (95% CI), (month)**	**median OS (95% CI), (month)**
Gou M [15]	30	54 (29–85)	NA	0		None	ORR, DCR	3.8 (2.8–4.8)	14.9 (7.6–21.7)
Antoniotti C [13]	NA	(18–75)	19.9 (IQR, 17.3–23.9)	67		FOLFOXIRI + Bevacizumab	PFS	12·9 (80% CI: 11·9–13·3)	NA
Xu YJ [16]	14	57.5 (27–73)	12	0		None	ORR, DCR	3.4 (2.2–4.6)	NA
Morano F [14]	17	58 (IQR, 53–65)	23.1 (IQR, 14.9–24.6)	0		None	ORR, DCR	7	18.4
Mettu NB [17]	NA	NA	20.9	41		Capecitabine + Bevacizumab	ORR, PFS	4.4 (4.1–6.4)	NA
Rahma OE [11]	NA	64 (36–79)	8.2	0		None	ORR, DCR	2.5 (0.6–3.3)	3.3 (0.6–3.4)
Kim RD [18]	NA	NA	NA	0		None	ORR, DCR	4.3 (2.3–7.9)	11.1 (9.7-NR)
Redman JM [19]	11	NA	NA	10		mFOLFOX6 + Bevacizumab	ORR, PFS, OS	10.1 (3.6–16.1)	15.1 (5.4–NR)
Fukuoka S [20]	18	55 (31–77)	NA	0		None	ORR	7.9 (2.9-NR)	NR (9.8-NR)
Eng C [10]	NA	NA	7.3 (IQR, 3.7–13.6)	80		Rego	ORR, PFS, OS	NA	NA
	NA	NA		80		Rego	ORR, PFS, OS	NA	NA
Kawazoe A [21]	17	63 (25–79)	6.3 (1.1–15.4)	0		None	ORR, DCR	1.6 (1.4–2.1)	7.3 (5.3–11.8)
Ren C [22]	3	54 (40–66)	NA	0		None	ORR, DCR	1.83 (1.80–1.86)	7.8 (0–17.07)
Kawazoe A [23]	12	61 (32–77)	NA	0		None	ORR	3.2 (2.8–4.4)	13.5 (8.2–15.1)
Parikh AR [12]	22	59 (26–83)	NA	0		None	ORR, DCR	2.5 (2.3–2.8)	10.9 (6.7–15.0)
			NA	0		None	ORR, DCR	NA	NA
Wang C [24]	16	60 (43–79)	NA	1		None	ORR, DCR	2	NR
Cousin S [25]	35	62 (26–83)	NA	0		None	ORR, DCR	3.6 (1.8–5.4)	10.8 (5.9–NR)
Wang C [26]	5	54	NA	0		None	ORR, DCR	NA	NA
Li J [27]	16	50 (33–73)	7.9 (6.5–9.3)	0		None	ORR, DCR	3.1 (2.32–3.89)	NA
Hellmann MD [28]	NA	NA	4.2 (0.7–40.2)	0		None	ORR	NA	NA
Kim DW [29]	16	59 (24–73)	NA	0		None	ORR, DCR	1.4 (1.4–1.5)	6.6 (4.3–12.2)
Patel MR [30]	9	56.5 (40–70)	NA	0		None	ORR, DCR	2.2 (1.8–6.0)	2.8 (1.8–5.1)
Bordonaro R [31]	5	64 (33–76)	NA	0		None	ORR, DCR	6 (2–8)	NR (6.5-NR)
Zhou H [32]	11	62 (43–78)	11.5 (10.3–12.7)	0		None	ORR, DCR	NA	NA
Yu W [33]	15	53.6 (mean)	NA	0		None	ORR, DCR	3.8	NA
Sun L [34]	13	54.6 (mean)	6.2 (3.9–8.43)	0		None	ORR, DCR	NA	NA
	14	53.0 (mean)		0		None	ORR, DCR	NA	NA
Jiang FE [35]	11	54 (31–72)	NA	0		None	ORR, DCR	NA	NA
O'Neil BH [36]	NA	NA	NA	0		None	ORR, DCR	NA	NA
Yarchoan M [37]	6	58 (44–85)	NA	0		None	ORR, DCR	2.7 (1.6–3.2)	7.1 (6.0–14.7)
Taylor K [38]	9	56 (36–78)	4.7	0		None	ORR, DCR	NA	NA
Martinelli E [39]	NA	NA	19.5 (12.8–22.8)	0		None	ORR, DCR	3.6 (3.3–3.9)	11.6 (8.3–15.0)
Wang C [40]	54	55 (IQR, 49–64)	NA	0		None	ORR, DCR	NA	NA
Lee JJ [41]	17	61 (30–79)	NA	0		None	ORR, DCR	2.1 (1.8–2.8)	6.2 (3.5–8.7)
Fang X [42]	18	60 (45–75)	NA	0		None	ORR, DCR	NA	NA
Bocobo AG [43]	14	55 (36–77)	NA	0		None	ORR, DCR	NA	NA
Huyghe N [44]	NA	NA	NA	0		None	ORR, DCR	NA	NA
			NA	0		None	ORR, DCR	NA	NA

The details of included studies can be found in the Table S2

Abbreviations: *ICIs* immune checkpoint inhibitors, *No* number, *NR* not reach, *NA* not available, *PFS* progression-free survival, *OS* overall survival, *CI* confidence interval, *RCT* randomized controlled trial, *MC* multicenter, *OL* open-label, *SA* single-arm, *DB* double-blin, *Pembr*o Pembrolizumab, *Atezo* Atezolizumab, *Nivo* Nivolumab, *Durva* Durvalumab, *Ave* Avelumab, *Camre* Camrelizumab, *Treme* Tremelimumab, *PD-1* programmed cell death-1, *PD-L1* Programmed cell death-Ligand 1, *NE* not evaluable, *ORR* objective response rate, *DCR* disease control rate, *mo* months, *Rego* regorafenib, *Sin* sintilimab, *Tori* toripalimab, *Aza* azacitidine, *pMMR* proficient mismatch repair, *MSI-H* microsatellite instability-high, *MSS* microsatelite stable, *MSI-L* microsatellite instability-low

### Data analysis

#### The efficacy of ICIs-based regimens for pMMR/non-MSI-H mCRC

##### ORR

**Fig. 2:**
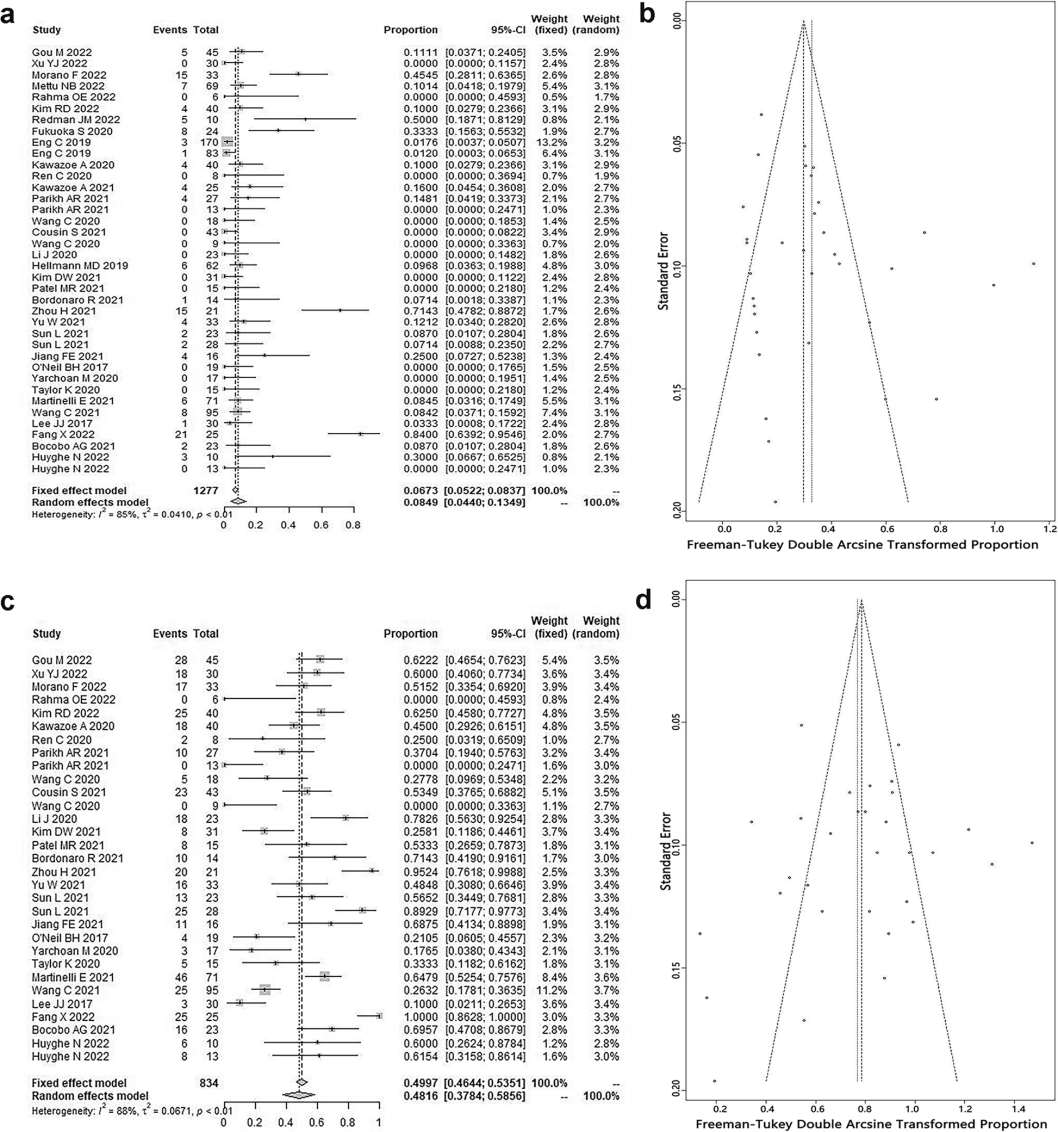
The pooled objective response rate (ORR) of immune checkpoint inhibitors (ICIs) in proficient mismatch repair (pMMR)/non-microsatellite instability-high (non-MSI-H) metastatic colorectal cancer (mCRC): **a** forest plot and **b** funnel plot; and the pooled disease control rate (DCR) of ICIs in pMMR/non-MSI-H mCRC: **c** forest plot and **d** funnel plot

A total of 38 cohorts containing 1277 patients were included to investigate the ORR of ICIs-based regimens for pMMR/non-MSI-H mCRC. Overall, the pooled ORR was 8.5% (95% CI: 4.4%-13.5%) (Fig. 2a), with 74.2% (95% CI: 61.4%-85.4%) as first-line regimen and 6.4% (95% CI: 3.3%-10.4%) as second-line or beyond regimen (Table 2), and without publication bias by funnel plot (Fig. 2b) and Egger’s test (z = 0.9, *P* = 0.39).

**Table 2:** The details of pooled ORR and DCR

ORR/DCR	Pooled rate (95% CI), %	No. of cohorts	*I*^*2*^ (95% CI), %	*P* for *I*^*2*^	Model	Egger's Test
ORR for ICIs-based therapy	8.5 (4.4–13.5)	38	84.5 (79.7–88.2)	< 0.01	Random effect	z = 0.9, *p*-value = 0.39
ORR for ICIs-based therapy as first-line	74.2 (61.4–85.4)	3	48.5 (0.0–85.0)	0.14	Fixed effect	z = -1.6, *p*-value = 0.12
ORR for ICIs-based therapy as second-line or beyond	6.4 (3.3–10.4)	28	66.7 (50.5–77.6)	< 0.01	Random effect	z = -0.4, *p*-value = 0.71
ORR-anti-CTLA-4 + anti-PD-(L)1 + radiotherapy	9.0 (0.9–21.9)	2	43.8	0.18	Fixed effect	/
ORR-anti-PD-(L)1 + anti-VEGF + chemotherapy	42.4 (10.0–78.6)	5	94.4 (89.8–97.0)	< 0.01	Random effect	z = 0.5, *p*-value = 0.62
ORR-anti-PD-(L)1 + anti-VEGF + chemotherapy (first-line)	74.2 (61.4–85.4)	3	48.5 (0.0–85.0)	0.14	Fixed effect	z = -1.6, *p*-value = 0.12
ORR-anti-PD-(L)1 + anti-VEGF + chemotherapy (second-line or beyond)	9.5 (4.0–16.7)	2	0.0	0.96	Fixed effect	/
ORR-anti-PD-(L)1 + anti-EGFR + chemotherapy	9.5 (0.0–53.2)	2	80.3 (15.3–95.4)	0.02	Random effect	/
ORR-anti-PD-(L)1 + TKIs	6.1 (1.7–12.4)	12	69.3 (44.3–83.1)	< 0.01	Random effect	z = 0.2, *p*-value = 0.87
ORR-anti-PD-(L)1 + chemotherapy	4.3 (0.6–10.1)	5	27.4 (0.0–71.4)	0.24	Fixed effect	z = -0.3, *p*-value = 0.79
ORR-anti-PD-(L)1 monotherapy	2.7 (0.0–9.4)	3	66.6 (0.0–90.4)	0.05	Random effect	z = -0.5, *p*-value = 0.60
DCR for ICIs-based therapy	48.2 (37.8–58.6)	31	87.9 (83.9–90.9)	< 0.01	Random effect	z = -1.2, *p*-value = 0.23
DCR for ICIs-based therapy as first-line	98.7 (92.0–100.0)	2	18.2	0.27	Fixed effect	/
DCR for ICIs-based therapy as second-line or beyond	45.1 (34.4–56.0)	26	84.8 (78.8–89.1)	< 0.01	Random effect	z = -1.4, *p*-value = 0.16
DCR-anti-CTLA-4 + anti-PD-(L)1 + radiotherapy	14.9 (0.0–63.6)	2	85.7 (42.7–96.4)	< 0.01	Random effect	/
DCR-anti-PD-(L)1 + anti-VEGF + chemotherapy	92.0 (68.3–100.0)	3	83.7 (51.0–94.6)	< 0.01	Random effect	z = -0.5, *p*-value = 0.60
DCR-anti-PD-(L)1 + anti-VEGF + chemotherapy (first-line)	98.7 (92.0–100.0)	2	18.2	0.27	Fixed effect	/
DCR-anti-PD-(L)1 + anti-VEGF + chemotherapy (second-line or beyond)	69.6 (49.0–87.0)	1	/	/	/	/
DCR-anti-PD-(L)1 + anti-EGFR + chemotherapy	60.9 (39.3–80.7)	2	0.0	0.94	Fixed effect	/
DCR-anti-PD-(L)1 + TKIs	59.8 (49.4–69.6)	11	66.9 (37.7–82.5)	< 0.01	Random effect	z = -0.5, *p*-value = 0.64
DCR-anti-PD-(L)1 + chemotherapy	39.5 (12.4–70.2)	4	84.9 (62.4–93.9)	< 0.01	Random effect	z = 1.8, *p*-value = 0.07
DCR-anti-PD-(L)1 monotherapy	25.1 (17.3–33.7)	2	0.0	0.70	Fixed effect	/

Abbreviations: *CI* confidence interval, *PD-(L)1* Programmed cell death-(Ligand) 1, *ORR* objective response rate, *DCR* disease control rate, *CTLA-4* cytotoxic T lymphocyte-associated antigen-4, *VEGF* vascular endothelial growth factor, *EGFR* epidermal growth factor receptor, *TKIs* tyrosine kinase inhibitors

Subgroup analysis was implemented based on various ICIs-based regimens (Table 2). The pooled ORR of ICIs monotherapy was low (ORR = 2.7%, 95% CI: 0.0%-9.4%), while that of ICIs plus anti-vascular endothelial growth factor (VEGF) agent and chemotherapy was high (ORR = 42.4%, 95% CI: 10.0%-78.6%), especially as first-line therapy.

There was no statistical difference on ORR for ICIs-based regimens in both RAS wild type (wt) and RAS mutant type (mt) pMMR/non-MSI-H mCRC (Fig. S1, OR = 1.4, 95% CI: 0.6–3.1, *P* = 0.46).

##### DCR

A total of 31 cohorts involving 834 patients were included to report the DCR of ICIs-based regimens for pMMR/non-MSI-H mCRC. Generally, the pooled DCR was 48.2% (95% CI: 37.8%-58.6%) (Fig. 2c), with 98.7% (95% CI: 92.0%-100.0%) as first-line regimen and 45.1% (95% CI: 34.4%-56.0%) as second-line or beyond regimen (Table 2), and without publication bias by funnel plot (Fig. 2d) and Egger’s test (z = -1.2, *P* = 0.23).

Subgroup analysis was carried out based on various ICIs-based regimens (Table 2). Obviously, the pooled DCR of ICIs plus anti-VEGF agent and chemotherapy was the best (DCR = 92.0%, 95% CI: 68.3%-100.0%), especially when it served as first-line therapy.

There was also no significantly difference on DCR for ICIs-based regimens in both RASwt and RASmt pMMR/non-MSI-H mCRC (Fig. S1, OR = 0.9, 95% CI: 0.5–1.9, *P* = 0.81).

##### OS

**Fig. 3:**
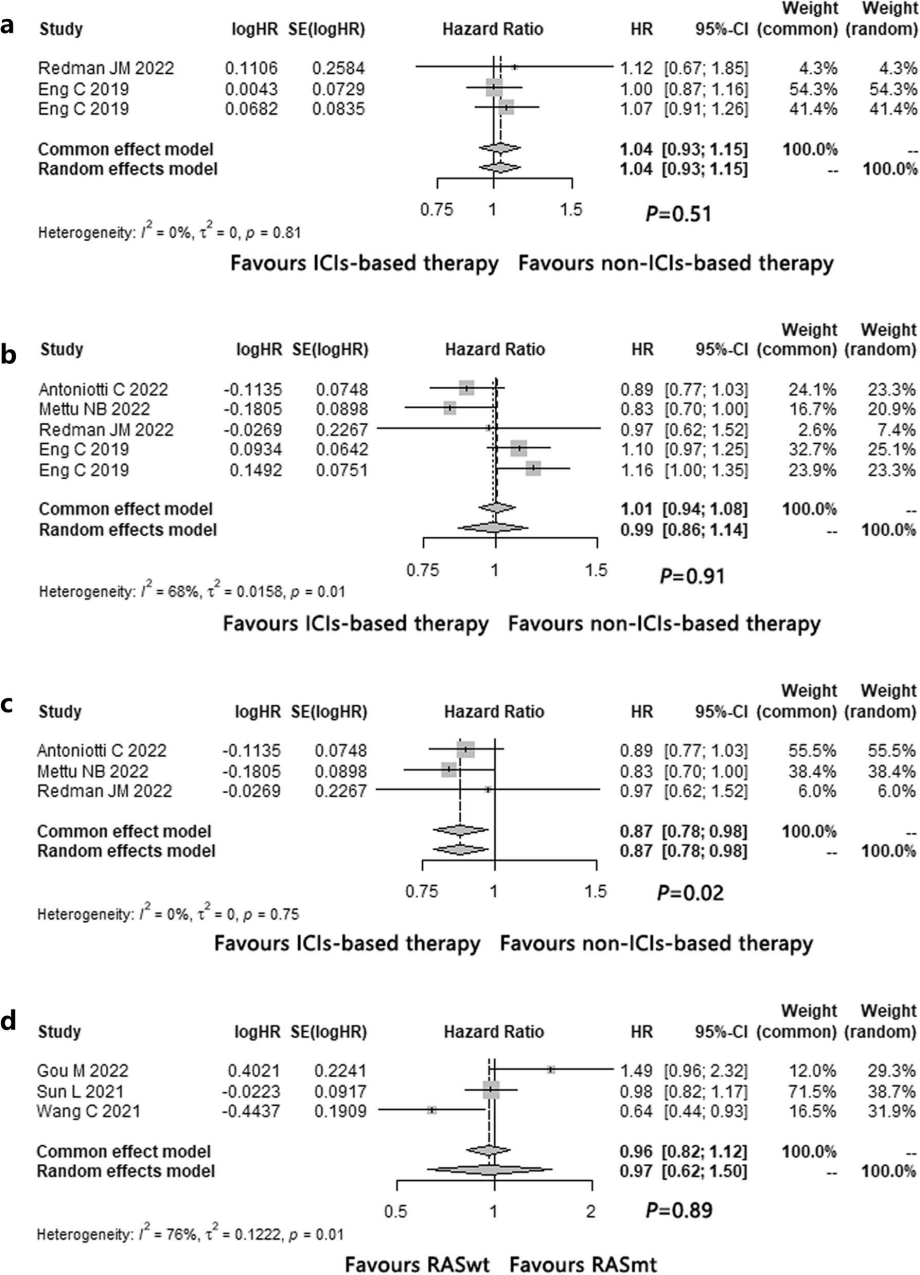
The forest plot of **a** overall survival (OS) and **b** progression-free survival (PFS) for ICIs-based versus non-ICIs-based therapy in pMMR/non-MSI-H mCRC; the forest plot of PFS for **c** ICIs plus anti-VEGF agent and chemotherapy versus non-ICIs-based therapy, and **d** RAS wild type (wt) versus RAS mutant type (mt) in pMMR/non-MSI-H mCRC

The pooled HR of OS for ICIs-based therapy versus non-ICIs-based therapy in pMMR/non-MSI-H mCRC was 1.0 (95% CI: 0.9–1.2, *P* = 0.51) (Fig. 3a) without publication bias through funnel plots (Fig. S2) and Egger’s test (z = 0.5, *P* = 0.60).

As a whole, the 12-month OS rate of ICIs-based therapy was 52.3% (95% CI: 46.4%-58.1%) (Table 3). According to subgroup analysis, the regimen of ICIs plus anti-VEGF agent and chemotherapy showed the highest 12-month OS rate (71.4%, 95% CI: 50.0%-89.1%) (Table 3).

**Table 3:** The details of pooled 6-month PFS rate and 12-month OS rate

	**Subgroups**	**Pooled rate (95% CI), %**	**No. of cohorts**	***I***^***2***^** (95% CI), %**	***P* for *I***^***2***^	**Model**	**Egger's Test**
6-month PFS rate	ICIs based-therapy	32.8 (23.5–42.7)	20	79.4 (68.8- 86.4)	< 0.01	Random effect	z = -0.1, *p*-value = 0.95
	anti-PD-(L)1 + chemotherapy	23.1 (9.6–39.7)	2	0.0	0.99	Fixed effect	/
	anti-PD-(L)1 + TKIs	29.3 (17.0–43.2)	10	80.5 (65.1- 89.1)	< 0.01	Random effect	z = -1.1, *p*-value = 0.28
	anti-PD-(L)1 + anti-VEGF + chemotherapy	55.2 (24.8–83.8)	3	80.7 (39.4- 93.8)	< 0.01	Random effect	z = 0.5, *p*-value = 0.60
	anti-PD-(L)1 + anti-EGFR + chemotherapy	39.1 (19.3–60.7)	2	0.0	0.94	Fixed effect	/
	First-line	71.5 (53.5–86.8)	2	0.0	0.37	Fixed effect	/
	Second-line or beyond	30.4 (21.5–40.0)	17	77.0 (63.5–85.5)	< 0.01	Random effect	z = -0.5, *p*-value = 0.62
12-month OS rate	ICIs based-therapy	52.3 (46.4–58.1)	12	36.0 (0.0- 67.7)	0.10	Fixed effect	z = -0.1, *p*-value = 0.89
	anti-PD-(L)1 + TKIs	52.1 (42.9–61.3)	5	0.0 (0.0- 76.2)	0.48	Fixed effect	z = 0.3, *p*-value = 0.80
	anti-PD-(L)1 + anti-VEGF + chemotherapy	71.4 (50.0–89.1)	1	/	/	/	/
	anti-PD-(L)1 + anti-EGFR + chemotherapy	47.8 (26.9–69.1)	2	0.0	0.86	Fixed effect	/
	First-line	71.4 (50.0–89.1)	1	/	/	/	/
	Second-line or beyond	51.2 (45.1–57.3)	10	33.0 (0.0–68.1)	0.14	Fixed effect	z = 0.0, *p*-value = 1.00

Abbreviations: *PFS* progression-free survival, *OS* overall survival, *CI* confidence interval, *PD-(L)1* Programmed cell death-(Ligand) 1, *CTLA-4* cytotoxic T lymphocyte-associated antigen-4, *VEGF* vascular endothelial growth factor, *EGFR* epidermal growth factor receptor, *TKIs* tyrosine kinase inhibitors

##### PFS

The pooled HR of PFS for ICIs-based therapy versus non-ICIs-based therapy in pMMR/non-MSI-H mCRC was 1.0 (95% CI: 0.9–1.1, *P* = 0.91) (Fig. 3b) without publication bias through funnel plots (Fig. S2) and Egger’s test (z = -0.5, *P* = 0.62).

Subgroup analysis was performed based on various ICIs-based regimens. Compared with non-ICIs-based therapy, the addition of ICIs to anti-VEGF agent plus chemotherapy brought significantly longer PFS for pMMR/non-MSI-H mCRC (HR = 0.9, 95% CI: 0.8–1.0, *P* = 0.02, Fig. 3c) without publication bias (funnel plots: Fig. S2, Egger’s test: z = 0.5, *P* = 0.60).

Subgroup analysis was also conducted based on RAS status. There was no significantly difference on PFS for ICIs-based regimens in both RASwt and RASmt pMMR/non-MSI-H mCRC (HR = 1.0, 95% CI: 0.6–1.5, *P* = 0.89, Fig. 3d), without publication bias (funnel plots: Fig. S2, Egger’s test: z = 0.5, *P* = 0.60).

As a whole, the 6-month PFS rate of ICIs-based therapy was 32.8% (95% CI: 23.5%-42.7%) (Table 3). According to subgroup analysis, the regimen of ICIs plus anti-VEGF agent and chemotherapy showed the highest 6-month PFS rate (55.2%, 95% CI: 24.8%-83.8%) (Table 3).

#### The safety of ICIs-based therapy in pMMR/non-MSI-H mCRC

A total of 21 cohorts were included to calculate the safety of ICIs-based therapy in pMMR/non-MSI-H mCRC (Table 4), and the pooled rate of grade 3 or beyond AEs was 31.8% (95% CI: 20.1%-44.8%). Despite the regimen of ICIs plus anti-VEGF agent and chemotherapy revealed higher incidence of grade 3 or beyond AEs, no additional treatment related adverse events (TRAEs) were observed.

**Table 4:** The pooled AEs

AEs	Pooled rate (95% CI), %	No. of study	*I*^*2*^ (95% CI), %	*P* for *I*^*2*^	Model	Egger's Test
ICIs-based therapy	31.8 (20.1–44.8)	21	89.5 (85.4- 92.5)	< 0.01	Random effect	z = -0.1, *p*-value = 0.95
anti-CTLA-4 + anti-PD-(L)1 + radiotherapy	29.3 (0.0–99.2)	2	95.2 (85.8- 98.4)	< 0.01	Random effect	/
anti-PD-(L)1 + TKIs	21.7 (9.4–37.0)	11	88.1 (80.7- 92.7)	< 0.01	Random effect	z = 0.0, *p*-value = 1.0
anti-PD-(L)1 + anti-VEGF + chemotherapy	60.1 (7.6–100.0)	2	93.0 (76.8- 97.9)	< 0.01	Random effect	/
anti-PD-(L)1 + chemotherapy	58.0 (17.8–93.1)	3	91.3 (77.5- 96.6)	< 0.01	Random effect	z = 1.6, *p*-value = 0.12

Abbreviations: *CI* confidence interval, *PD-(L)1* Programmed cell death-(Ligand) 1, *AE* Adverse events, *CTLA-4* cytotoxic T lymphocyte-associated antigen-4, *VEGF* vascular endothelial growth factor, *EGFR* epidermal growth factor receptor, *TKIs* tyrosine kinase inhibitors

### Assessment of study quality

The quality assessment of 35 recruited articles was summarized in Table S3 with relatively satisfying results for bias risk assessment.

## Discussion

In the last decade, ICIs has initiated a new era for immunotherapy in oncology by monoclonal antibodies to release the anti-tumor activity of preexisting tumor-specific T-cell immunity, which inspired researchers to focus on the application of ICIs in mCRC. However, a lot of studies have confirmed that ICIs monotherapy has not shown significant clinical activity in pMMR/non-MSI-H mCRC, which was considered with an immune-desert or immune-excluded (or “cold”) microenvironment. Therefore, accumulating studies have been carried out recently focusing on ICIs-based combination regimens in which ICIs have been incorporated with chemotherapy, radiotherapy and anti-VEGF agent in order to transform immunologically “cold” pMMR/non-MSI-H mCRC into responsive “hot” lesions. However, the results of such studies have been inconsistent [9–14] and the AEs caused by ICIs cannot be ignored. To overcome the limitations of individual studies, we performed a meta-analysis of relevant trials to investigate the benefit and safety of ICIs-based therapy for pMMR/non-MSI-H mCRC.

Based on the existing studies, the pooled results of our study revealed that the addition of ICIs into anti-VEGF agent plus chemotherapy (especially first-line) is promising in pMMR/non-MSI-H mCRC in terms of ORR, DCR, PFS, 6-monhs PFS rate and 12-month OS rate. At the same time, it has been supported that the potential clinical efficacy of anti-VEGF agent plus ICIs combination was also founded in other tumors such as HCC and NSCLC. For HCC, compared with sorafenib monotherapy, atezolizumab plus bevacizumab (IMbrave 150), and Sintilimab plus bevacizumab (ORIENT-32) were founded to significantly improve PFS and OS [45, 46]; with regard to NSCLC, the addition of atezolizumab to bevacizumab plus chemotherapy (IMpower150) significantly improved PFS and OS among patients with metastatic nonsquamous NSCLC, regardless of PD-L1 expression and EGFR or ALK genetic alteration status [47], Sintilimab plus bevacizumab biosimilar IBI305 and chemotherapy (ORIENT-31) improved PFS of patients with EGFR-mutated non-squamous NSCLC who progressed on EGFR tyrosine-kinase inhibitor therapy [48]. As we know, ICIs can effectively alleviate immune escape [49] and activate the human immune system to kill tumor cells, aims to improve immunity and enhance the anti-tumor response, then to achieve its anti-tumor effects [50, 51]. The limited advantage may be attributed to that cancer with pMMR/non-MSI-H has an immune-desert or immune-excluded (or “cold”) microenvironment, finally resulting in a blunted immune activation of tumor microenvironment that causes the futility of ICIs in these patients [52]. It has been recorded that cytotoxic agents are able to induce immunogenic cell death and activate CD8+ T lymphocytes, favoring an immune enriched microenvironment as the consequence of the release of tumor-associated neoantigens [53]. However, there are a lot of neovascularization with special structure in tumor tissue, which makes it difficult for antitumor drugs and immune cells to reach the tumor site. The VEGF pathway plays a pivotal role in establishing and maintaining an immunosuppressive tumor microenvironment. Therefore, the addition of anti-VEGF agent has a consistent vessel fortification effect in pMMR/non-MSI-H cancer, and can establish an immune permissive tumor microenvironment [54]. Therefore, the combination of chemotherapy, antiangiogenic and ICIs might have subadditivity, additivity or synergism effects to delays progression in patients achieving tumor shrinkage with subsequent release of neoantigens and immune activation of tumor microenvironment that allows ICIs efficacy [55–57].

Although there was no difference between ICI-based therapy and non-ICI-based therapy in both OS and PFS of pMMR/non-MSI-H mCRC on the whole, the subgroup analysis revealed that the addition of ICIs to anti-VEGF agent plus chemotherapy could significantly improve PFS of pMMR/non-MSI-H mCRC; moreover, there was no directly correlation between the improvement of PFS and RAS status. At the same time, some other advantages of ICI-based therapy were still founded among these studies. The study conducted by Eng et al. found that although not superior to standard therapy, treatment with the combination of ICIs and MEK inhibitor resulted in equivalent survival without the introduction of any new AEs [10]. Besides, Redman et al. found that despite a lack of improvement in clinical outcomes in the experimental arm, the addition of ICIs to chemotherapy was biologically active and produced multifunctional T-cell responses to cascade antigens MUC1 and brachyury [19].

With regard to the safety, the regimens of ICIs plus chemotherapy with/without anti-VEGF agent revealed higher incidence of grade 3 or beyond AEs. Among the included studies, Bocobo et al. found that the grade 3 or beyond TRAEs only occurred in 28% patients, of which less than half (11%) were immune-related and none was associated with bevacizumab [43]; besides, Redman et al. revealed that no TRAEs were observed outside the expected safety profile with the addition of ICIs to bevacizumab plus chemotherapy, and most TRAEs were chemotherapy-related and controllable [19].

The best strategy and biomarkers of ICIs for pMMR/non-MSI-H mCRC remain to be established. On one hand, in order to seek the best strategy of ICIs-based therapy for pMMR/non-MSI-H mCRC, we are obliged to optimize which regimen is beneficial in combination with ICIs (with maximizing efficacy and minimizing toxicity), facilitate clinical research based on biomarkers, and explore the development of other ICIs drugs and cell-based treatment schemes [58]; on the other hand, in screening the beneficiaries of ICIs for pMMR/non-MSI-H mCRC, we need to further seek appropriate biomarkers (such as TMB, PD-L1 expression, tumor infiltrating lymphocytes (TILs), and status of polymerase ε (POLE), neutrophil to lymphocyte ratio (NLR), and platelet-lymphocyte ratio (PLR)) [59–62]. Moreover, prospective, larger confirmatory and translational studies are recommended in the future.

## Limitations

This study came up with three drawbacks as follows: firstly, there were only 4 RCTs, despite containing 39 cohorts 1723 patients, for analyzing the efficacy and safety of ICIs in pMMR/ non-MSI-H mCRC; secondly, considering the limited number of studies with survival outcomes for ICIs in pMMR/non-MSI-H mCRC patients, we took the ORR and DCR as primary endpoints; finally, only studies published in English were included, which might yield language bias to some degree.

## Conclusions

ICIs-based combination therapy, especially the addition of ICIs to first-line anti-VEGF agent plus chemotherapy, is promising in pMMR/non-MSI-H mCRC with good efficacy and controllable toxicity.

### Supplementary Information

**Additional file 1:** **Table S1.** PRISMA Checklist

**Additional file 2:** **Table S2.** The list of the included studies.

**Additional file 3:** **Table S3.** The Newcastle-Ottawa scale for quality assessment of the studies

**Additional file 4:** **Figure S1. **The pooled objective response rate (ORR) of immune checkpoint inhibitors (ICIs)-based therapy in RAS wild type (wt) versus RAS mutant type (mt) proficient mismatch repair (pMMR)/non-microsatellite instability-high (non-MSI-H) metastatic colorectal cancer (mCRC): (a) forest plot and (b) funnel plot; the pooled disease control rate (DCR) of ICIs-based therapy in RASwt versus RASmt pMMR/non-MSI-H mCRC: (c) forest plot and (d) funnel plot.

**Additional file 5:** **Figure S2. **The funnel plot of (a) overall survival (OS) and (b) progression-free survival (PFS) for ICIs-based therapy versus non-ICIs-based therapy in pMMR/non-MSI-H mCRC; the funnel plot of PFS for (c) ICIs plus anti-VEGF agent and chemotherapy versus non-ICIs-based therapy, and (d) RAS wild type (wt) versus RAS mutant type (mt) in pMMR/non-MSI-H mCRC.

## Data Availability

The datasets generated during and/or analyzed during the current study are available from the corresponding author on reasonable request.

## Abbreviations

ICIs: Immune checkpoint inhibitors
No: Number
NR: Not reach
NA: Not available
PFS: Progression-free survival
OS: Overall survival
CI: Confidence interval
RCT: Randomized controlled trial
MC: Multicenter
OL: Open-label
SA: Single-arm
Pembro: Pembrolizumab
Atezo: Atezolizumab
Nivo: Nivolumab
Durva: Durvalumab
Ave: Avelumab
Camre: Camrelizumab
PD-1: Programmed cell death 1
PD-L1: Programmed cell death-ligand 1
ORR: Objective response rate
DCR: Disease control rate
mo: Months
Rego: Regorafenib
Sin: Sintilimab
Tori: Toripalimab
Aza: Azacitidine
AEs: Adverse events
pMMR: Proficient mismatch repair
MSI-H: Microsatellite instability-high
CRC: Colorectal cancer
MSS: Microsatelite stable
MSI-L: Microsatellite instability-low
TILs: Tumor infiltrating lymphocytes
POLE: Polymerase ε
NLR: Neutrophil to lymphocyte ratio
PLR: Platelet-lymphocyte ratio
TRAEs: Treatment related adverse events
VEGF: Vascular endothelial growth factor
NSCLC: Non-small cell lung cancer
TNBC: Triple negative breast cancer
HCC: Hepatocellular carcinoma
TMB: Tumor mutational burden
OR: Odd ratio
HR: Hazard ratio
