# Supplementary material for: Efficacy and safety of immune checkpoint inhibitors in Proficient Mismatch Repair (pMMR)/ Non-Microsatellite Instability-High (non-MSI-H) metastatic colorectal cancer: a study based on 39 cohorts incorporating 1723 patients
Source: BMC Immunol. 2023 Sep 1;24:27. doi: 10.1186/s12865-023-00564-1 (PMC10472580; doi:10.1186/s12865-023-00564-1)
Supplement: Supplementary file 2 — Additional file 2: Table S2. The list of the included studies. [file 12865_2023_564_MOESM2_ESM.docx]

**Table S2 The list of the included studies**

| **First author** | **Title** | **Journal** | **Year** | **Volume** | **Issue** | **Page** |
| --- | --- | --- | --- | --- | --- | --- |
| Gou M ^15^ | Fruquintinib in Combination With PD-1 Inhibitors in Patients With Refractory Non-MSI-H/pMMR Metastatic Colorectal Cancer: A Real-World Study in China. | Front Oncol | 2022 | 12 | / | 851756 |
| Antoniotti C ^13^ | Upfront FOLFOXIRI plus bevacizumab with or without atezolizumab in the treatment of pa tients with metastatic colorectal cancer (AtezoTRIBE): a multicentre, open-label, randomised, controlled, phase 2 trial. | Lancet Oncol | 2022 | 23 | 7 | 876-887 |
| Xu YJ ^16^ | Regorafenib combined with programmed cell death-1 inhibitor against refractory colorectal cancer and the platelet-to-lymphocyte ratio's prediction on effectiveness. | World J Gastrointest Oncol | 2022 | 14 | 4 | 920-934 |
| Morano F ^14^ | Temozolomide Followed by Combination With Low-Dose Ipilimumab and Nivolumab in Patients With Microsatellite-Stable, O^6^-Methylguanine-DNA Methyltransferase-Silenced Metastatic Colorectal Cancer: The MAYA Trial. | J Clin Oncol | 2022 | 40 | 14 | 1562-1573 |
| Mettu NB ^17^ | Assessment of Capecitabine and Bevacizumab With or Without Atezolizumab for the Treatment of Refractory Metastatic Colorectal Cancer: A Randomized Clinical Trial. | JAMA Netw Open | 2022 | 5 | 2 | e2149040 |
| Rahma OE ^11^ | Phase IB study of ziv-aflibercept plus pembrolizumab in patients with advanced solid tumors. | J Immunother Cancer | 2022 | 10 | 3 | e003569 |
| Kim RD ^18^ | A phase I/Ib study of regorafenib and nivolumab in mismatch repair proficient advanced refractory colorectal cancer. | Eur J Cancer | 2022 | 169 |  | 93-102 |
| Redman JM ^19^ | [A Randomized Phase II Trial of mFOLFOX6 + Bevacizumab Alone or with AdCEA Vaccine + Avelumab Immunotherapy for Untreated Metastatic Colorectal Cancer.](https://pubmed.ncbi.nlm.nih.gov/35274710/) | Oncologist | 2022 | 27 | 3 | 198-209 |
| Fukuoka S ^20^ | Regorafenib Plus Nivolumab in Patients With Advanced Gastric or Colorectal Cancer: An Open-Label, Dose-Escalation, and Dose-Expansion Phase Ib Trial (REGONIVO, EPOC1603). | J Clin Oncol | 2020 | 38 | 18 | 2053-2061 |
| Eng C ^10^ | Atezolizumab with or without cobimetinib versus regorafenib in previously treated metastatic colorectal cancer (IMblaze370): a multicentre, open-label, phase 3, randomised, controlled trial. | Lancet Oncol | 2019 | 20 | 6 | 849-861 |
| Kawazoe A ^21^ | Multicenter Phase I/II Trial of Napabucasin and Pembrolizumab in Patients with Metastatic Colorectal Cancer (EPOC1503/SCOOP Trial). | Clin Cancer Res | 2020 | 26 | 22 | 5887-5894 |
| Ren C ^22^ | Anti-PD-1 antibody SHR-1210 plus apatinib for metastatic colorectal cancer: a prospective, single-arm, open-label, phase II trial. | Am J Cancer Res | 2020 | 10 | 9 | 2946-2954 |
| Kawazoe A ^23^ | TAS-116 (Pimitespib), an Oral HSP90 Inhibitor, in Combination with Nivolumab in Patients with Colorectal Cancer and Other Solid Tumors: An Open-Label, Dose-Finding, and Expansion Phase Ib Trial (EPOC1704). | Clin Cancer Res | 2021 | 27 | 24 | 6709-6715 |
| Parikh AR ^12^ | Radiation therapy enhances immunotherapy response in microsatellite stable colorectal and pancreatic adenocarcinoma in a phase II trial. | Nat Cancer | 2021 | 2 | 11 | 1124-1135 |
| Wang C ^24^ | Regorafenib and Nivolumab or Pembrolizumab Combination and Circulating Tumor DNA Response Assessment in Refractory Microsatellite Stable Colorectal Cancer. | Oncologist | 2020 | 25 | 8 | e1188-e1194 |
| Cousin S ^25^ | Regorafenib-Avelumab Combination in Patients with Microsatellite Stable Colorectal Cancer (REGOMUNE): A Single-arm, Open-label, Phase II Trial. | Clin Cancer Res | 2021 | 27 | 8 | 2139-2147 |
| Wang C ^26^ | A Pilot Feasibility Study of Yttrium-90 Liver Radioembolization Followed by Durvalumab and Tremelimumab in Patients with Microsatellite Stable Colorectal Cancer Liver Metastases. | Oncologist | 2020 | 25 | 5 | 382-e776 |
| Li J ^27^ | The Efficacy and Safety of Regorafenib in Combination With Anti-PD-1 Antibody in Refractory Microsatellite Stable Metastatic Colorectal Cancer: A Retrospective Study. | Front Oncol | 2020 | 10 | / | 594125 |
| Hellmann MD ^28^ | Phase Ib study of atezolizumab combined with cobimetinib in patients with solid tumors. | Ann Oncol | 2019 | 30 | 7 | 1134-1142 |
| Kim DW ^29^ | A phase 1/2 trial of ibrutinib in combination with pembrolizumab in patients with mismatch repair proficient metastatic colorectal cancer. | Br J Cancer | 2021 | 124 | 11 | 1803-1808 |
| Patel MR ^30^ | A phase 2 trial of trifluridine/tipiracil plus nivolumab in patients with heavily pretreated microsatellite-stable metastatic colorectal cancer. | Cancer Med | 2021 | 10 | 4 | 1183-1190 |
| Bordonaro R ^31^ | Trifluridine/tipiracil in combination with oxaliplatin and either bevacizumab or nivolumab in metastatic colorectal cancer: a dose-expansion, phase I study. | ESMO Open | 2021 | 6 | 5 | 100270 |
| Zhou H ^32^ | Preliminary Efficacy and Safety of Camrelizumab in Combination With XELOX Plus Bevacizumab or Regorafenib in Patients With Metastatic Colorectal Cancer: A Retrospective Study. | Front Oncol | 2021 | 11 | / | 774445 |
| Yu W ^33^ | Efficacy and Safety of Regorafenib Combined with Toripalimab in the Third-Line and beyond Treatment of Advanced Colorectal Cancer. | J Oncol | 2021 | 2021 | / | 9959946 |
| Sun L ^34^ | Efficacy and Safety of Fruquintinib Plus PD-1 Inhibitors Versus Regorafenib Plus PD-1 Inhibitors in Refractory Microsatellite Stable Metastatic Colorectal Cancer. | Front Oncol | 2021 | 11 | / | 754881 |
| Jiang FE ^35^ | Efficacy and safety of regorafenib or fruquintinib plus camrelizumab in patients with microsatellite stable and/or proficient mismatch repair metastatic colorectal cancer: an observational pilot study. | Neoplasma | 2021 | 68 | 4 | 861-866 |
| O'Neil BH ^36^ | Safety and antitumor activity of the anti-PD-1 antibody pembrolizumab in patients with advanced colorectal carcinoma. | PLoS One | 2017 | 12 | 12 | e0189848 |
| Yarchoan M ^37^ | A phase 2 study of GVAX colon vaccine with cyclophosphamide and pembrolizumab in patients with mismatch repair proficient advanced colorectal cancer. Cancer Med. |  | 2020 | 9 | 4 | 1485-1494 |
| Taylor K ^38^ | An open-label, phase II multicohort study of an oral hypomethylating agent CC-486 and durvalumab in advanced solid tumors. | J Immunother Cancer | 2020 | 8 | 2 | e000883 |
| Martinelli E ^39^ | Cetuximab Rechallenge Plus Avelumab in Pretreated Patients With RAS Wild-type Metastatic Colorectal Cancer: The Phase 2 Single-Arm Clinical CAVE Trial. . 2021;7(10):1529-1535. doi: 10.1001/jamaoncol.2021.2915. | JAMA Oncol | 2021 | 7 | 10 | 1529-1535 |
| Wang C ^40^ | Clinical Response to Immunotherapy Targeting Programmed Cell Death Receptor 1/Programmed Cell Death Ligand 1 in Patients With Treatment-Resistant Microsatellite Stable Colorectal Cancer With and Without Liver Metastases. | JAMA Netw Open | 2021 | 4 | 8 | e2118416 |
| Lee JJ ^41^ | Phase 2 study of pembrolizumab in combination with azacitidine in subjects with metastatic colorectal cancer. | J Clin Oncol | 2017 | 35 | 15_suppl | 3054 |
| Fang X ^42^ | A phase 2 trial of sintilimab (IBI 308) in combination with CAPEOX and bevacizumab (BBCAPX) as first-line treatment in patients with RAS-mutant, microsatellite stable, unresectable metastatic colorectal cancer. | J Clin Oncol | 2022 | 40 | 16_suppl | 3563 |
| Bocobo AG ^43^ | Phase II study of pembrolizumab plus capecitabine and bevacizumab in microsatellite stable (MSS) metastatic colorectal cancer (mCRC). | J Clin Oncol | 2021 | 39 | 3_suppl | 77 |
| Huyghe N ^44^ | Interim analysis of the phase II AVETUXIRI trial: Avelumab combined with cetuximab and irinotecan for treatment of refractory microsatellite stable (MSS) metastatic colorectal cancer (mCRC). | J Clin Oncol | 2022 | 40 | 16_suppl | 3595 |
