# Supplementary material for: Efficacy and safety of immune checkpoint inhibitors in Proficient Mismatch Repair (pMMR)/ Non-Microsatellite Instability-High (non-MSI-H) metastatic colorectal cancer: a study based on 39 cohorts incorporating 1723 patients
Source: BMC Immunol. 2023 Sep 1;24:27. doi: 10.1186/s12865-023-00564-1 (PMC10472580; doi:10.1186/s12865-023-00564-1)
Supplement: Supplementary file 3 — Additional file 3: Table S3. The Newcastle-Ottawa scale for quality assessment of the studies [file 12865_2023_564_MOESM3_ESM.docx]

**Table S3 The Newcastle-Ottawa scale for quality assessment of the studies**

| Included study | Selection | | | | Comparability | Exposure/outcome | | | Total |
| --- | --- | --- | --- | --- | --- | --- | --- | --- | --- |
|  | Is the case definition adequate? /ascertainment of exposure | Representativeness of the cases/ exposed cohort | Selection of controls/the non-exposed cohort | Definition of controls/demonstration that outcome of interest was not present at start of study | Comparability of both groups/ cohorts on the basis of the design or analysis | Ascertainment of exposure/ assessment of outcome | same method of ascertainment for both groups/ was follow-up long enough for outcomes to occur | Non-response rate/ adequacy of follow up of cohorts | scores |
| Gou M 2022 | * | * | * | - | * | * | * | * | 7 |
| Antoniotti C 2022 | * | * | * | * | ** | * | * | * | 9 |
| Xu YJ 2022 | * | * | * | - | * | * | * | * | 7 |
| Morano F 2022 | * | * | * | * | * | * | * | * | 8 |
| Mettu NB 2022 | * | * | * | * | ** | * | * | * | 9 |
| Rahma OE 2022 | * | * | * | * | * | * | * | * | 8 |
| Kim RD 2022 | * | * | * | * | * | * | * | * | 8 |
| Redman JM 2022 | * | * | * | * | ** | * | * | * | 9 |
| Fukuoka S 2020 | * | * | * | * | * | * | * | * | 8 |
| Eng C 2019 | * | * | * | * | ** | * | * | * | 9 |
| Kawazoe A 2020 | * | * | * | * | * | * | * | * | 8 |
| Ren C 2020 | * | * | * | * | * | * | * | * | 8 |
| Kawazoe A 2021 | * | * | * | * | * | * | * | * | 8 |
| Parikh AR 2021 | * | * | * | * | * | * | * | * | 8 |
| Wang C 2020 | * | * | * | - | * | * | * | * | 7 |
| Cousin S 2021 | * | * | * | * | * | * | * | * | 8 |
| Wang C 2020 | * | * | * | * | * | * | * | * | 8 |
| Li J 2020 | * | * | * | - | * | * | * | * | 7 |
| Hellmann MD 2019 | * | * | * | * | * | * | * | * | 8 |
| Kim DW 2021 | * | * | * | * | * | * | * | * | 8 |
| Patel MR 2021 | * | * | * | * | * | * | * | * | 8 |
| Bordonaro R 2021 | * | * | * | * | * | * | * | * | 8 |
| Zhou H 2021 | * | * | * | - | * | * | * | * | 7 |
| Yu W 2021 | * | * | * | - | * | * | * | * | 7 |
| Sun L 2021 | * | * | * | - | * | * | * | * | 7 |
| Jiang FE 2021 | * | * | * | - | * | * | * | * | 7 |
| O'Neil BH 2017 | * | * | * | * | * | * | * | * | 8 |
| Yarchoan M 2020 | * | * | * | * | * | * | * | * | 8 |
| Taylor K 2020 | * | * | * | * | * | * | * | * | 8 |
| Martinelli E 2021 | * | * | * | * | * | * | * | * | 8 |
| Wang C 2021 | * | * | * | - | * | * | * | * | 7 |
| Lee JJ 2017 | * | * | * | * | * | * | * | * | 8 |
| Fang X 2022 | * | * | * | * | * | * | * | * | 8 |
| Bocobo AG 2021 | * | * | * | * | * | * | * | * | 8 |
| Huyghe N 2022 | * | * | * | * | * | * | * | * | 8 |
