# Supplementary material for: Efficacy and safety of immune checkpoint inhibitors in Proficient Mismatch Repair (pMMR)/ Non-Microsatellite Instability-High (non-MSI-H) metastatic colorectal cancer: a study based on 39 cohorts incorporating 1723 patients
Source: BMC Immunol. 2023 Sep 1;24:27. doi: 10.1186/s12865-023-00564-1 (PMC10472580; doi:10.1186/s12865-023-00564-1)
Supplement: Supplementary file 4 — Additional file 4: Figure S1. The pooled objective response rate (ORR) of immune checkpoint inhibitors (ICIs)-based therapy in RAS wild type (wt) versus RAS mutant type (mt) proficient mismatch repair (pMMR)/non-microsatellite instability-high (non-MSI-H) metastatic colorectal cancer (mCRC): (a) forest plot and (b) funnel plot; the pooled disease control rate (DCR) of ICIs-based therapy in RASwt versus RASmt pMMR/non-MSI-H mCRC: (c) forest plot and (d) funnel plot. [file 12865_2023_564_MOESM4_ESM.docx]

**
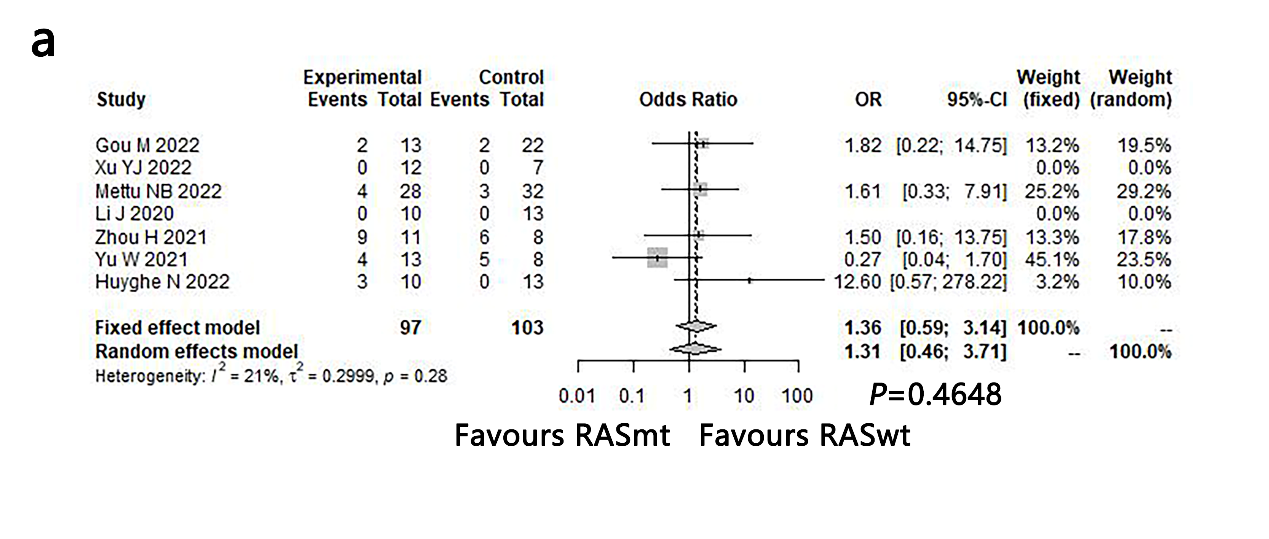

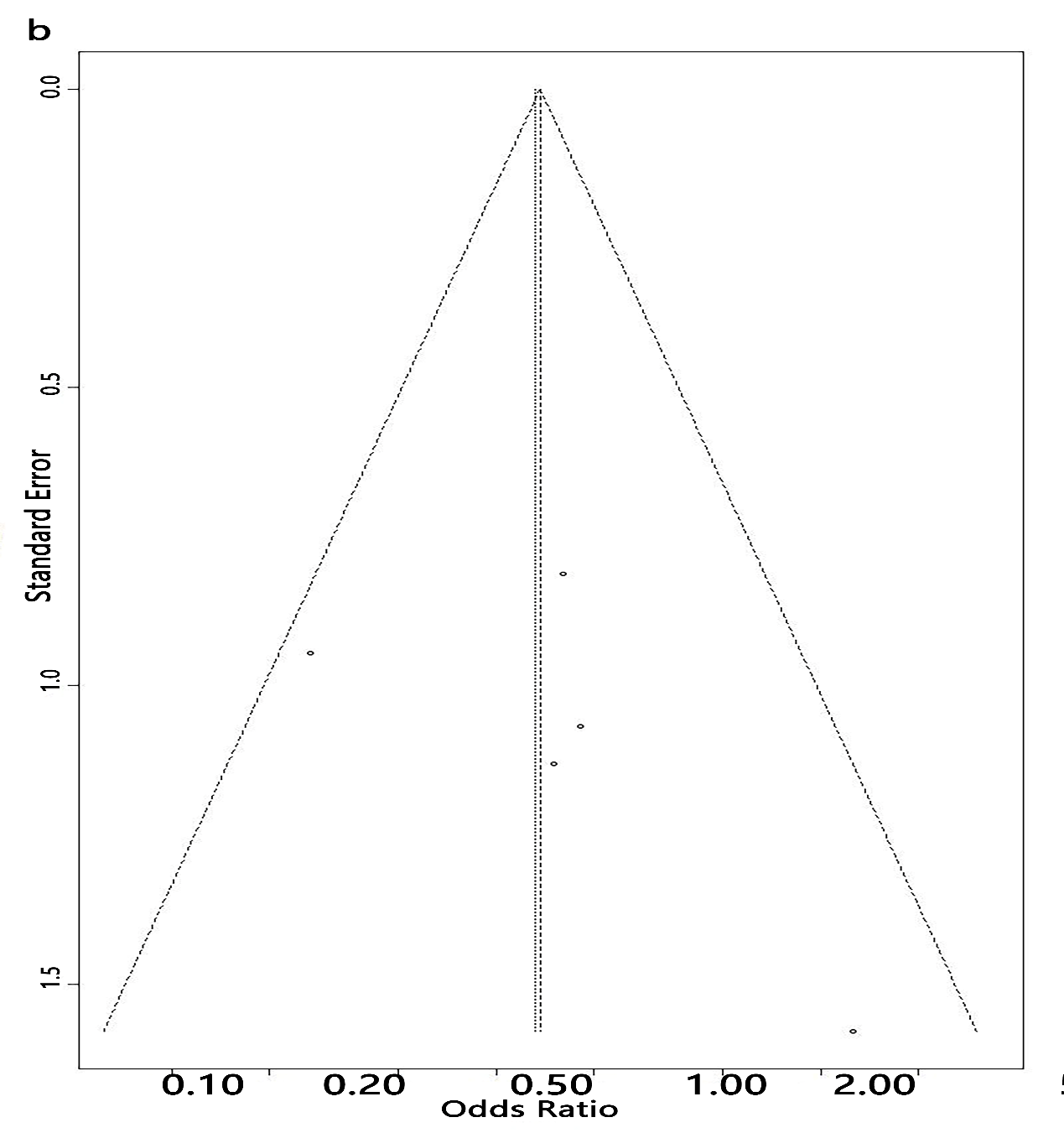

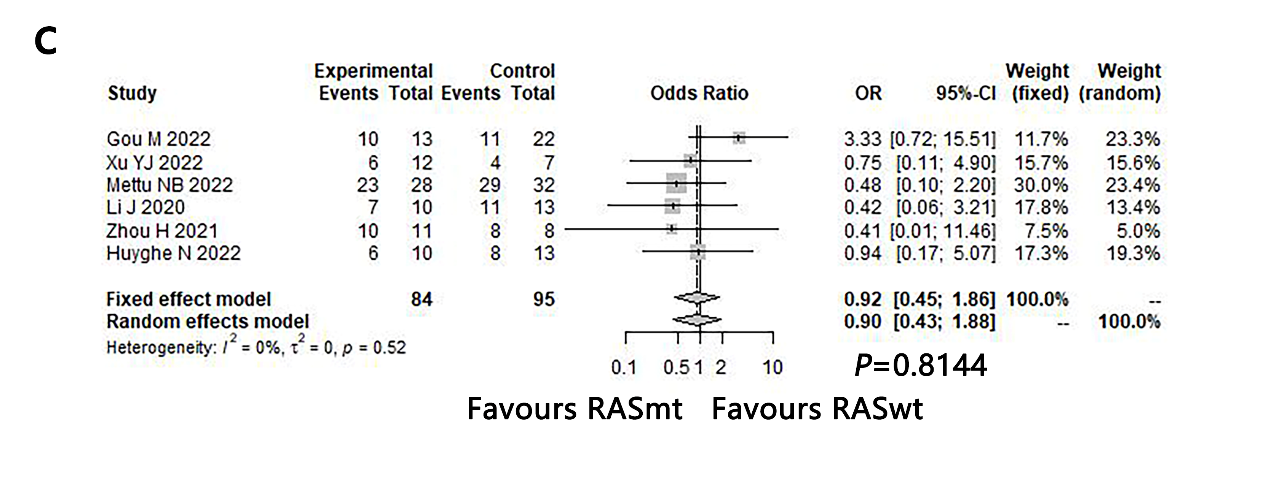

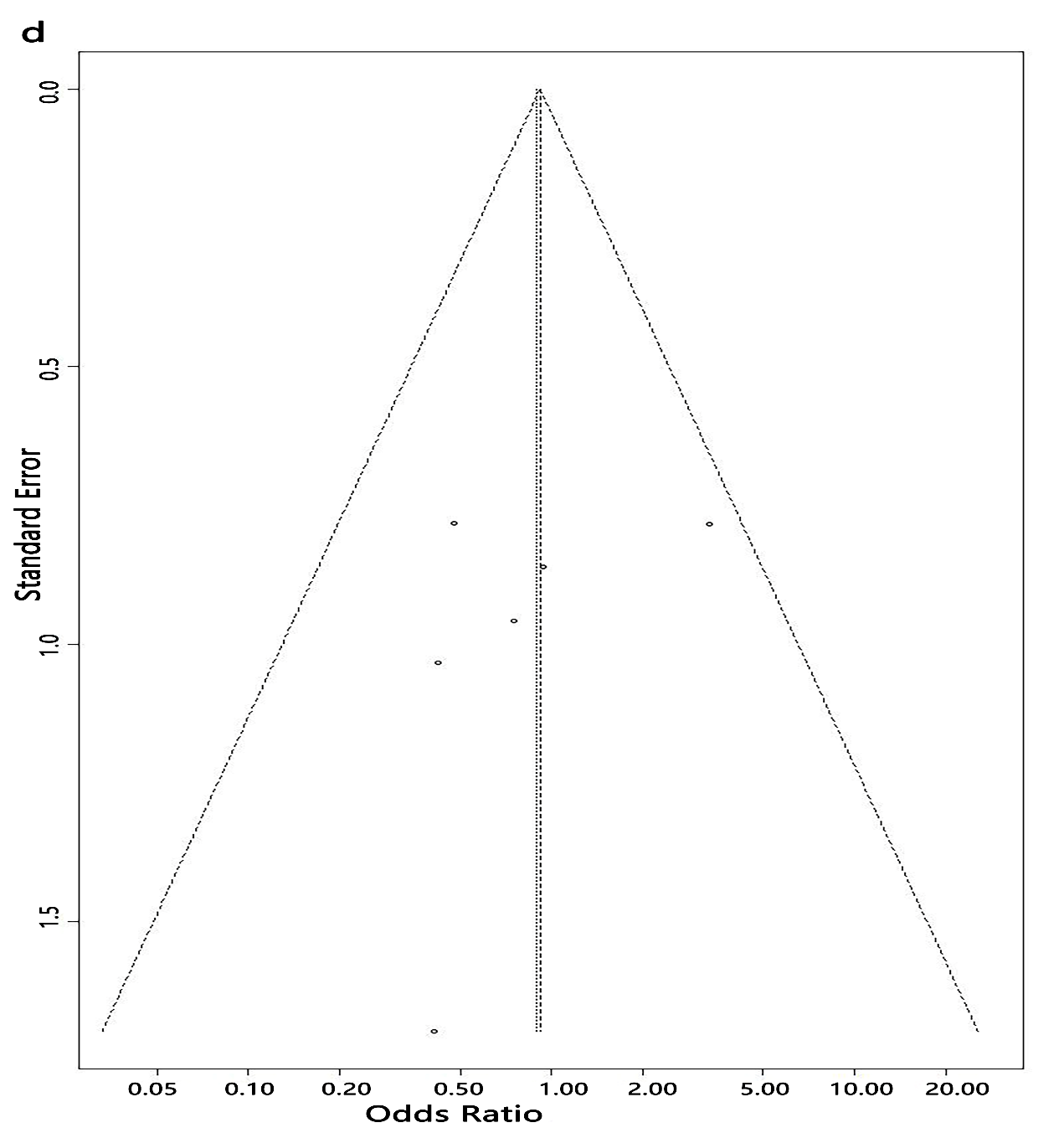
Figure S1** The pooled objective response rate (ORR) of immune checkpoint inhibitors (ICIs)-based therapy in RAS wild type (wt) versus RAS mutant type (mt) proficient mismatch repair (pMMR)/non-microsatellite instability-high (non-MSI-H) metastatic colorectal cancer (mCRC): (a) forest plot and (b) funnel plot; the pooled disease control rate (DCR) of ICIs-based therapy in RASwt versus RASmt pMMR/non-MSI-H mCRC: (c) forest plot and (d) funnel plot.
