# Supplementary material for: Efficacy and safety of immune checkpoint inhibitors in Proficient Mismatch Repair (pMMR)/ Non-Microsatellite Instability-High (non-MSI-H) metastatic colorectal cancer: a study based on 39 cohorts incorporating 1723 patients
Source: BMC Immunol. 2023 Sep 1;24:27. doi: 10.1186/s12865-023-00564-1 (PMC10472580; doi:10.1186/s12865-023-00564-1)
Supplement: Supplementary file 5 — Additional file 5: Figure S2. The funnel plot of (a) overall survival (OS) and (b) progression-free survival (PFS) for ICIs-based therapy versus non-ICIs-based therapy in pMMR/non-MSI-H mCRC; the funnel plot of PFS for (c) ICIs plus anti-VEGF agent and chemotherapy versus non-ICIs-based therapy, and (d) RAS wild type (wt) versus RAS mutant type (mt) in pMMR/non-MSI-H mCRC. [file 12865_2023_564_MOESM5_ESM.docx]

**
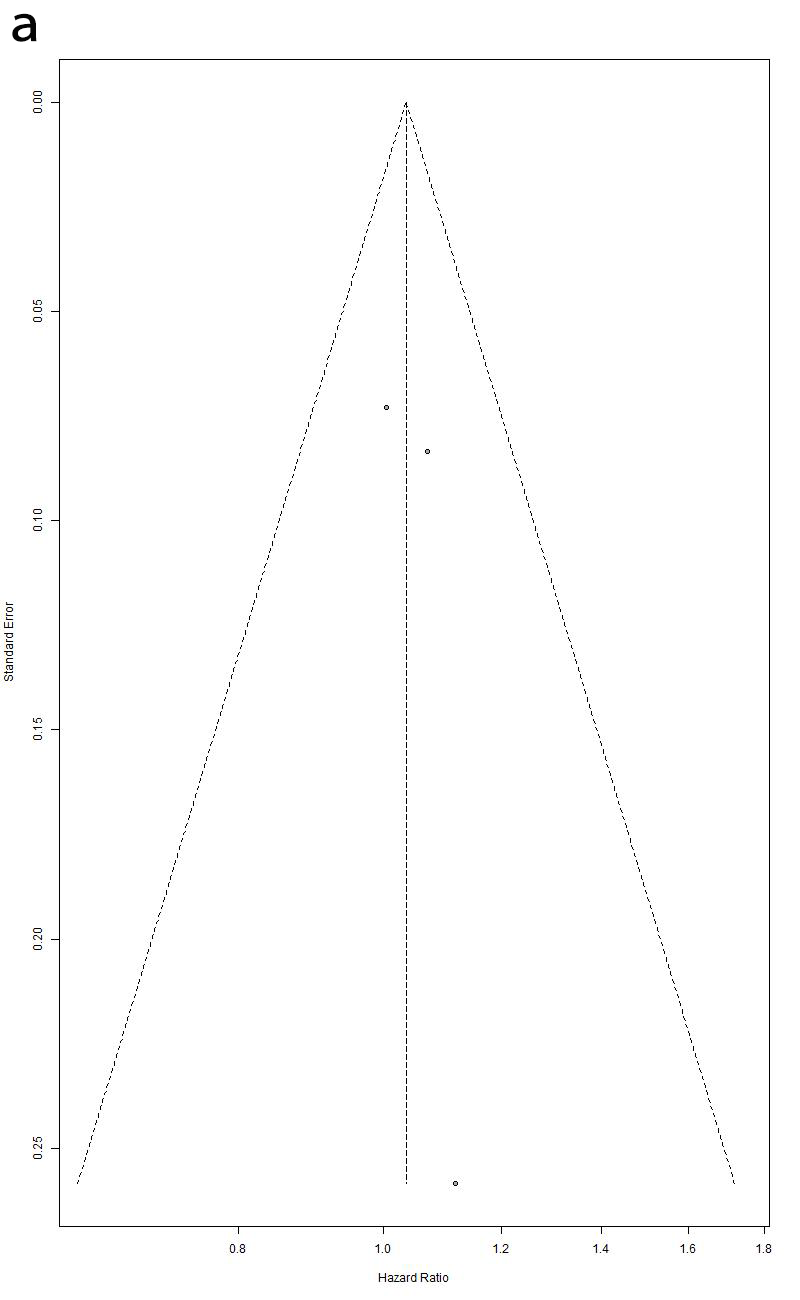
**

**
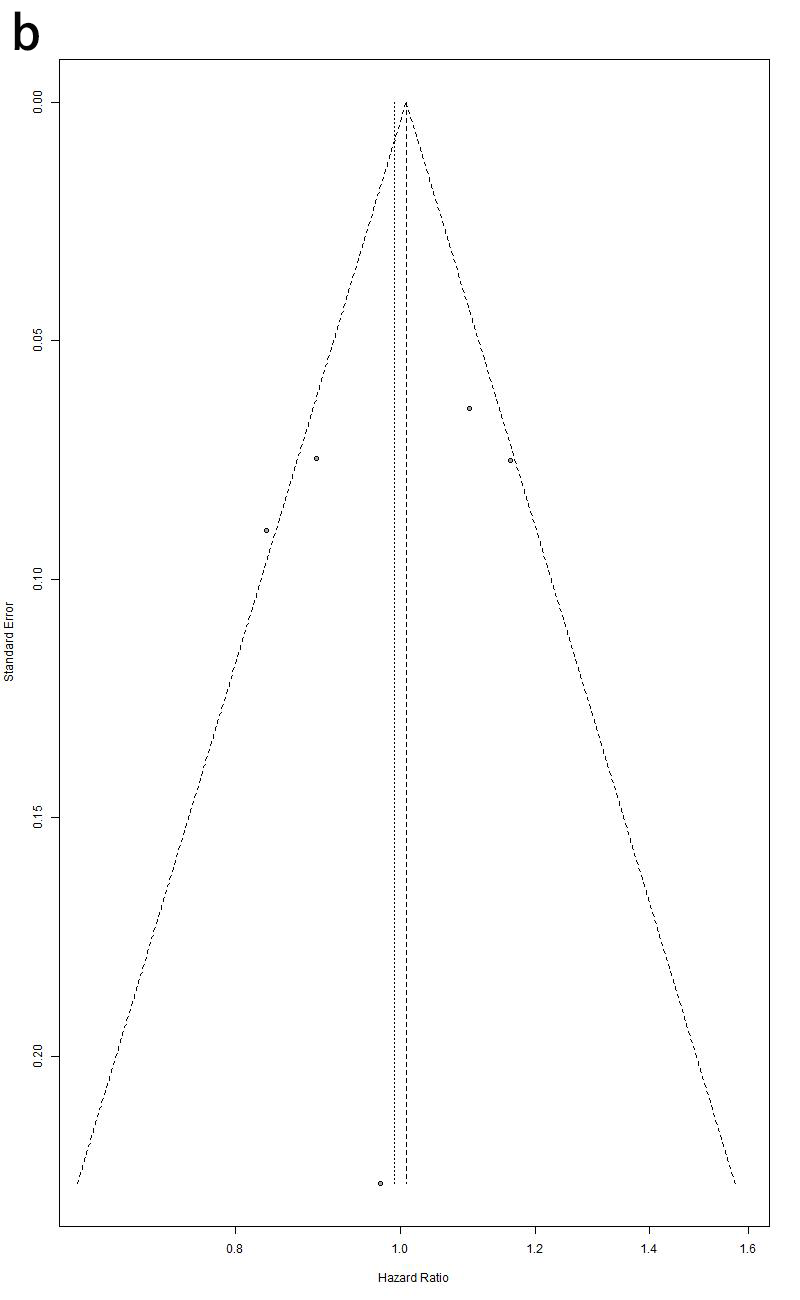
**

**
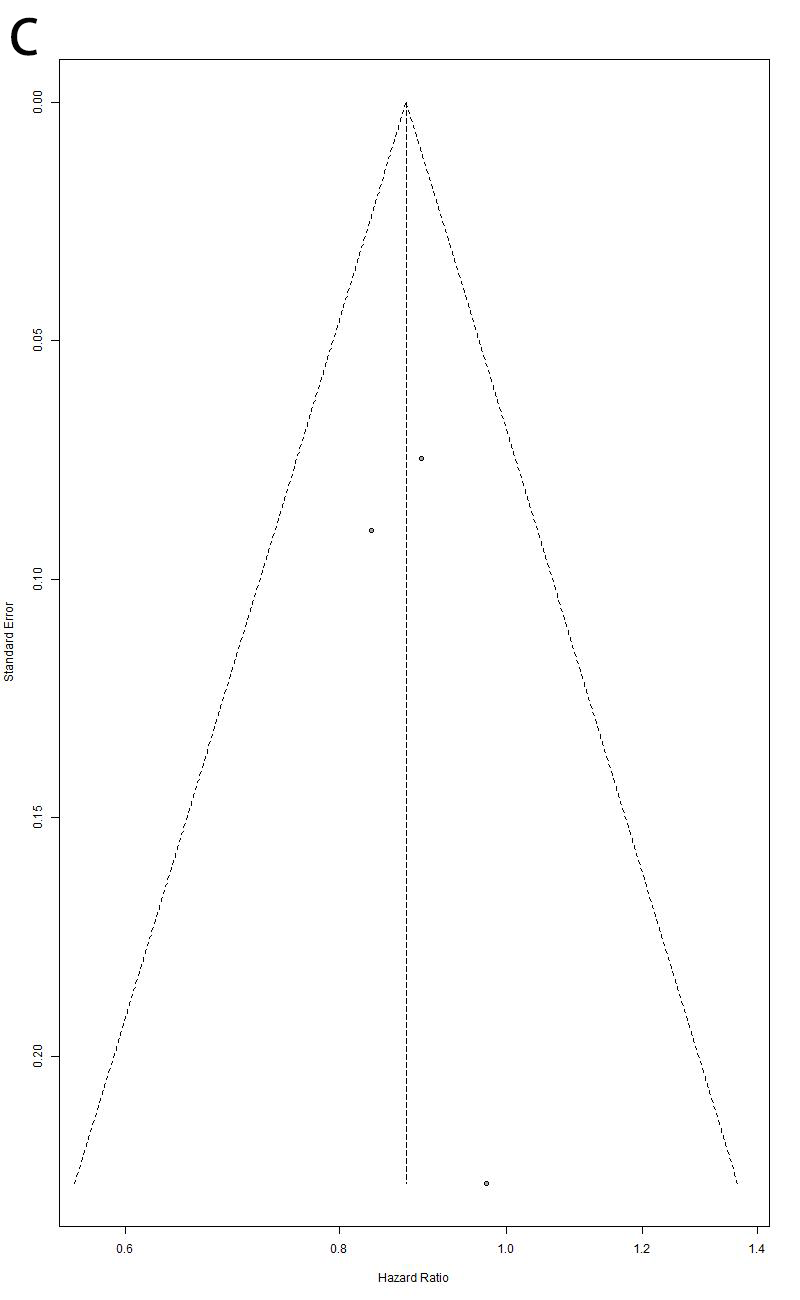
**

**
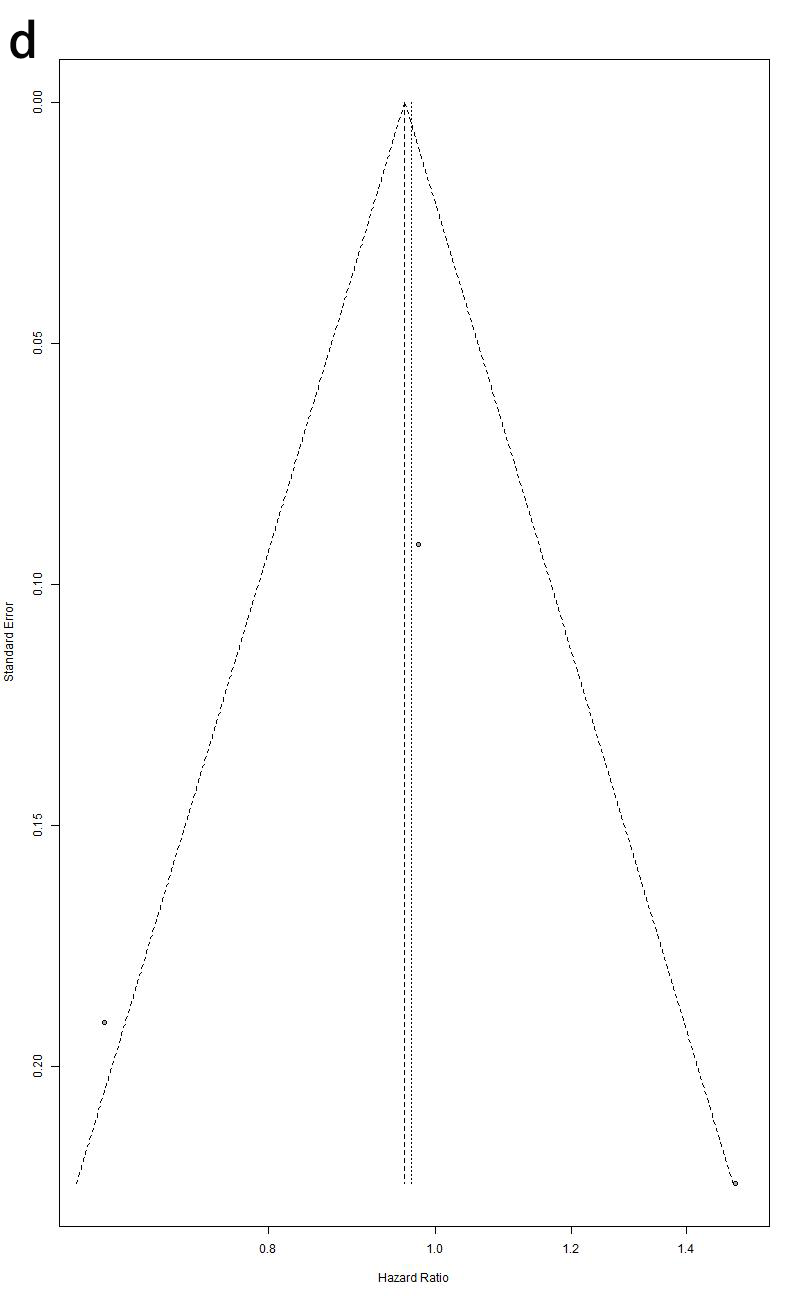
Figure S2** The funnel plot of (a) overall survival (OS) and (b) progression-free survival (PFS) for ICIs-based therapy versus non-ICIs-based therapy in pMMR/non-MSI-H mCRC; the funnel plot of PFS for (c) ICIs plus anti-VEGF agent and chemotherapy versus non-ICIs-based therapy, and (d) RAS wild type (wt) versus RAS mutant type (mt) in pMMR/non-MSI-H mCRC.
